# Effects of blood flow restriction training on muscle fitness and cardiovascular risk of obese college students

**DOI:** 10.3389/fphys.2023.1252052

**Published:** 2024-01-03

**Authors:** Yanhong Su, Fuqing Wang, Meng Wang, Shiyong He, Xiaolei Yang, Zhilin Luan

**Affiliations:** ^1^ Key Laboratory of Sports Human Science in Liaoning Province, College of Physical Education, Liaoning Normal University, Dalian, China; ^2^ Advanced Institute for Medical Sciences, Dalian Medical University, Dalian, China

**Keywords:** blood flow restriction training, heart rate variability, muscle fitness, neuromuscular activation, glucolipid metabolism, cardiovascular risk factors

## Abstract

**Purpose:** The aim of this study was to investigate the effect of blood flow restriction (BFR) combined with low-intensity resistance training (RT) on cardiovascular risk factors in obese individuals.

**Methods:** Twenty-six male obese college students were recruited and randomly assigned to a control group (CON, *n* = 8), a low-intensity RT group (RT, *n* = 9), and a combined BFR training and low-intensity RT group (BFRT, *n* = 9).

**Results:** The subjects in BFRT group showed significant reductions in body fat percentage and waist-to-hip ratio and a significant increase in lean mass and muscle mass; the peak torque, peak power, and endurance ratio of knee extensors and elbow flexors were significantly upregulated; the root mean square (RMS) for the medial femoral muscle, lateral femoral muscle and biceps significantly increased; the diastolic blood pressure (DBP) showed a significant decrease. The BFRT group also showed significant up-regulations in RMS of the difference between the adjacent R-R intervals (RMSSD), high-frequency power (HF) of parasympathetic modulatory capacity, the standard deviation of R-R intervals (SDNN) of overall heart rate variability (HRV) changes and low-frequency power (LF) of predominantly sympathetic activity. In addition, glycated hemoglobin (HbA1C), insulin resistance index (HOMA-IR) and fasting blood glucose (FBG) were all significantly downregulated in BFRT group. In parallel, low-density lipoprotein (LDL-C) significantly reduced while high-density lipoprotein (HDL-C) significantly increased in BFRT group.

**Conclusion:** BFR combined with low-intensity RT training effectively improved body composition index, increased muscle mass, improved neuromuscular activation, enhanced muscle strength and endurance, which in turn improved abnormal glucolipid metabolism and enhanced cardiac autonomic regulation.

## 1 Introduction

The prevalence of obesity has been reported to be increasing worldwide and is expected to reach 57.8% by 2030 (Tian et al., 2022). Accumulated data highlight the deleterious effects of obesity on the risk of cardiovascular disease (CVD), which is the leading cause of death and disability worldwide (Wormser et al., 2011; Yusuf and Anand, 2011; Khan et al., 2018). Obese people with high-fat mass in the abdominal region are those who contribute to the clustering of risk factors for CVD in overweight and obese populations (Powell-Wiley et al., 2021). Obesity causes increased oxidation of low-density lipoproteins, glucolipid abnormalities, reduced cardiac autonomic regulation and many other cardiovascular risk factors involved in the development of CVD (Grundy, 2016; Yadav et al., 2017). Autonomic dysfunction is accompanied by the development of hypertension and a reduction in heart rate variability (HRV), which substantially increases cardiovascular mortality (Yadav et al., 2017). Thus, alleviation of metabolic abnormalities and/or dysfunction of the cardiac autonomic nervous system in the obese state is critical for the prevention of CVD.

High adiposity and low muscle mass independently exacerbate the risk of CVD (Terada et al., 2022). Skeletal muscle is the largest regulator of metabolism in the body, and obesity leads to a reduction in intrinsic force-generating capacity or muscle mass of skeletal muscle (Tallis et al., 2021). The relationship between muscular health and CVD has received increasing attention in recent years. Relatively high muscle strength is negatively associated with triglyceride and total cholesterol levels and glucose levels (Lawman et al., 2016). This may be linked to skeletal muscle endocrine function and increased basal metabolic rate (Chen et al., 2020). Increased muscle mass may also lead to a greater hemodynamic response. In healthy pre-hypertensive adults, muscle strength and blood pressure are negatively correlated, suggesting that muscle strength may function as a protective factor against hypertension and thereby reduce the incidence of cardiovascular disease (Lopez-Jaramillo et al., 2022). Muscle fiber growth, on the other hand, is achieved primarily by stimulating large exercise loads to disrupt and reorganize them. During heavy-load resistance training (HLRT), mechanical strain is the primary stimulus for anabolic activity (Pearson and Hussain, 2015). In turn, excessive exercise loads are prone to muscle and joint injury with more than 90% of injuries typically associated with excessive loading parameters (Kerr et al., 2010).

Blood flow restriction (BFR) training typically involves low-intensity resistance exercise or aerobic exercise with blood flow restriction applied to the working limb using a pneumatic cuff or an elastic cuff. Resistance-based BFR training may increase skeletal muscle strength (Centner et al., 2019). In addition, BFR training is less load-bearing, generates less mechanical strain, and induces increased metabolic stress (Pearson and Hussain, 2015; Wang et al., 2023). The current American Heart Association recommends that the load of RT for the rehabilitation of patients with cardiovascular disease should be reduced (30% of the single maximal load for the upper extremity and 50%–60% of the single maximal load for the lower extremity), as this will not only lead to improved muscle strength and endurance but also not result in excessive increases in blood pressure or other adverse cardiovascular outcomes (Kambič et al., 2019). Healthy adults require higher training loads (>75% 1-repetition maximum [1-RM]) to achieve optimal improvements in muscular hypertrophy and strength (2009). In contrast, the recommended load for patients with coronary artery disease is much lower (<30% 1-RM) and insufficient to induce gains in isometric strength and hypertrophy capacity (Piepoli et al., 2011). The characteristics of BFR training suggest that BFR training combined with low-intensity RT is relatively safe for patients with cardiovascular disease (Kambič et al., 2019).

BFR training is also widely used among young people. Wilson and others conducted a study enrolled twenty male participants aged 21 ± 3 years with BFRT-30% 1RM resistance training. The participants completed a total of 5 testing sessions separated by a minimum of 72 h’ rest over a 2- to 3-week period, and the results showed that resistance training increases muscle size (Wilson et al., 2013). Kacin and Strazar (2011) has shown that BFRT can increase muscle size by designing a study including ten healthy males with BFRT-15% maximal voluntary muscle contraction [MVC], four sessions/week for 4 weeks. Manimmanakorn et al. (2013) (thirty well-trained netball players enrolled with BFRT-20% 1RM resistance training, one time/day for 5 days) and Yasuda et al. (2011) (forty young males enrolled ant divided into four groups respectively with 75% 1RM resistance training, BFRT-30% 1RM resistance training, 75% 1RM and BFRT-30% 1RM, non-training, three times/week for 6 weeks) demonstrated that BFRT has the effect of increasing muscle cross-sectional area. Studies in young obese individuals have shown that 12 weeks of cycling at 40% VO_2max_ combined with BFRT improves body composition and lipid metabolism levels (percent body fat, body mass index, and glucose (GLU), total cholesterol (TC), triglycerides, and low-density lipoprotein cholesterol (LDL-C)), promotes overall fitness, reduces body fat, and manages body weight in obese male college students (Chen et al., 2021). This is important for those who are unable to perform moderate to high intensity exercise to improve their health and athletic performance.

In healthy adults, BFR training combined with low-intensity RT can promote protein synthesis, increase muscle cross-sectional area, and improve muscle strength while reducing systemic cardiovascular adverse effects. However, the application of BFR training to obese people has not been extensively studied. Therefore, we investigated the effects of BFR training with low-intensity RT on low muscle mass and high adiposity production in obese people, as well as changes in cardiovascular risk factors such as abnormal glucolipid metabolism and reduced heart rate variability, through long-term BFR training combined with low-intensity RT as an intervention for obese people.

## 2 Materials and methods

### 2.1 Participants

According to the Chinese Guidelines for the Prevention and Control of Overweight and Obesity in School-aged Children and Adolescents, in the population aged >18 years, males with body fat percentage >20% are considered obese. In the present study, we enrolled twenty-six obese male college students between the ages of twenty and twenty-four who have previously never undergone professional physical exercise, and never used hormonal medications with no smoking habit.

Subjects were excluded if they did not complete the training in the expected experimental cycle; or if they were unable to complete the test due to the risk of pre-exercise screening for risk of certain acute and chronic illnesses; and whether they did not engage in training more than 2 times a week. Exercise group subjects were not permitted to participate in exercise training similar to the present study. Table 1 shows the baseline profile of subjects.

**TABLE 1:** Basic subjects’ information (M ± SD).

Group	N	Height (cm) baseline	Weight (kg)Baseline	BF(%) baseline	Muscle Mass (kg) baseline
CON	8	175.75 ± 3.37	76.13 ± 6.78	23.38 ± 2.91	53.40 ± 3.46
RT	9	172.89 ± 5.01	74.99 ± 5.49	23.81 ± 2.79	52.79 ± 3.08
BFRT	9	175.44 ± 4.43	78.28 ± 6.97	24.73 ± 2.89	54.42 ± 4.02

CON, control group; RT, low-intensity resistance group; BFRT, blood flow restriction combined with low-intensity resistance group; M, mean; SD, standard deviation; BF (%), body fat percentage.

The study followed the Declaration of Helsinki and was approved by the Ethics Committee of Liaoning Normal University (LL2021034). Participants were informed about the protocol and signed a written informed consent form before the start of the study. During the experiment, the participants experienced collective centralized training, no medical incident occurred, and no participant withdrawed.

Prior to the intervention, an independent samples *t*-test was used to assess the homogeneity of the physical characteristics of the subjects across the CON, RT and BFRT groups (Table 1). Homogeneity was ensured because there were no significant differences in height, weight, BF%, and Muscle Mass between the two groups (all *p >* 0.05).

### 2.2 Study design

**FIGURE 1:**
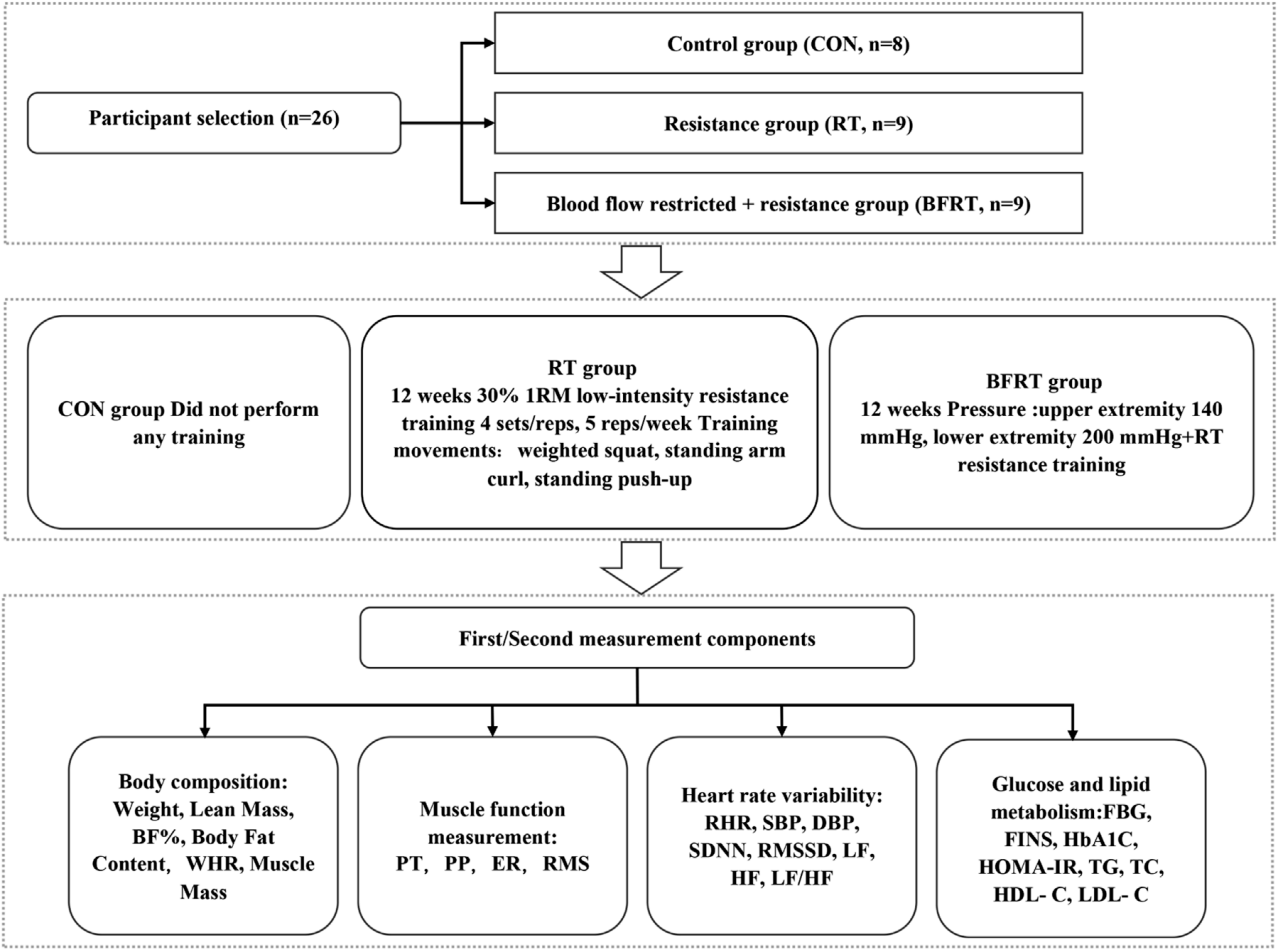
Experimental flow graph of the Study. Notes: BF (%), body fat percentage; WHR, waist-to-hip ratio; PT, peak torque; PP, peak power; ER, endurance ratio; RMS, root mean square amplitude; SBP, systolic blood pressure; DBP, diastolic blood pressure; RHR, quiet heart rate; SDNN, standard deviation of R-R intervals; RMSSD, root mean square of the difference between the adjacent R-R intervals; HF, high-frequency power; LF, low-frequency power; LF/HF, high-frequency power to low-frequency power; FBG, fasting blood glucose; FINS, fasting insulin; HbA1C, glycated hemoglobin; TC, total cholesterol; TG, triglycerides; HDL-C, high-density lipoprotein cholesterol; LDL-C, low-density lipoprotein cholesterol.

The CON group performed no training, the RT group performed a low-intensity 30% 1-RM resistance training, and the BFRT group performed a 30% 1RM of low-intensity pressure resistance training (upper limb pressure 140 mmHg, pressure 200 mmHg in the lower extremities) with weighted squats, standing arm curls, and push-ups in the standing position (4 sets/time, 5 times/week, 12 weeks). Subjects’ body composition, muscle function, glucose and lipid metabolism, and cardiovascular risk were measured prior to the first training session and following the final training session (Figure 1).

### 2.3 Exercise intervention

In this study, the following training protocol was set up by reviewing the relevant literature and according to the requirement of our own experimental design. The training movements, exercise intensity, exercise frequency, the interval between sets, and pressurization pressure in the study were set with reference to the corresponding studies (Kraemer et al., 1990; Loenneke et al., 2013; Fahs et al., 2015; Yang et al., 2022). The 1-RM values of standing arm curl, standing neck front push-up, and weighted deep squat were calculated for subjects in the RT and BFRT groups during training according to Holten’s table using the indirect test method of repetitive test of 10-RM, and the 1-RM values were measured centrally once every 4 weeks of training, and the load intensity of the subjects’ later exercise regimen was developed based on the 1-RM values. The intensity of the exercise was set at a uniform 30% 1-RM. Each exercise consisted of three movements including 2 upper limb movements and 1 lower limb movement, each movement was performed in 4 sets, 30 reps in the first set, 15 reps in the last 3 sets, the 60 s in the first set, 30 s in the last 3 sets, and 2s for each complete movement according to the rhythm. The exercise training was performed according to the above steps for both experimental groups. During the training, the subjects in RT group did not wear compression equipment while the ones in BFRT group wore compression equipment: 140 mmHg for the upper limb and 200 mmHg for the lower limb, with the pressure released between sets.

The program on athletic training is as follows: Table 2

**TABLE 2:** Resistance training program

Training process	Training content	Training time and number of sets
Preliminary	Subjects were instructed to perform a full-body warm-up in an organized manner. (Hip, knee, ankle exercises; trotting; high kicks; back kicks, etc.)	Each movement is performed for 10s; one set of each movement
Fundamental part	Subjects were instructed to perform 30% 1RM of full joint range of motion standing arm curls and standing anterior neck thrusts. Four sets of each movement were performed with a 30s interval between sets	Completion time per group/s (60:30:30:30)
	After upper body training, lower body training is performed	Repetitions per set/each (30:15:15:15)
	Subjects were instructed to perform 30% 1RM weighted squats in an orderly fashion for 4 sets with a 30s interval between sets	
Concluding part	Subjects, perform full body relaxation activities. (shoulder stretches, leg stretches, arm stretches, hip stretches, etc.)	Each movement is performed for 10s; one set of each movement

### 2.4 Muscle fitness and body composition

Body composition was measured in the Sports Human Science Laboratory of the School of Physical Education and Sport, Liaoning Normal University (8:00 a.m., fasting state), using a Korean DX-200 instrument, the test method: the subject first removed all metal products and stood barefoot on the electrode plate, the staff entered the relevant information, so that the subject held the electrodes in their hands, thumbs pressed down on the sensors, arms naturally drooped, and remained static, the progress bar rolled over. When the progress bar scrolls to the end, the body composition test is finished, click “test results” and print, the test is completed. The whole body fat, body fat rate, muscle mass and lean body mass were measured according to the experimental design.

The test is divided into two modes of testing, the first mode is the isometric muscle strength test, which tests the maximal muscular strength of the subject’s knee extensors and elbow flexors, and the test index chosen is the peak torque (PT) of the corresponding muscle group of the joint. The second mode of testing is centripetal-centripetal testing with 20 repetitions of 180°/s. In this mode, the fast strength and muscular endurance of the subject’s knee extensors and elbow flexors are reflected. The rapid strength test is based on peak power (PP) and the muscular endurance test is based on endurance ratio (ER): i.e., work done on the last 3 extensors (flexors)÷work done on the first 3 extensors (flexors). This test started with a knee isometric test for 5 s, with the knee flexion angle set to 100° (Steidl-Müller et al., 2018) and a 1- minute rest for a 180°/s centripetal-centripetal contraction pattern of the same joint. Isometric elbow test for 5 s, with the elbow joint set to a flexion angle of 90° (Talib et al., 2021) and a 1-min rest for a centripetal-centripetal contraction mode of 180°/s of the same joint. At least 3 trials were performed in each mode and the maximum value was taken.

### 2.5 Electromyography measurements

The test instrument is an ME6000 surface electromyography instrument made in Finland. The electromyography test is conducted simultaneously with the 5s maximum autonomous (MVC) isometric muscle strength test for knee and elbow joints, and the electromyography is observed during the test to ensure the maximum force is generated. During the test, the lower limb muscle groups were the medial and lateral femoral muscles and the upper limb muscle groups were the biceps. Surface electromyographic signals (sEMG) data were acquired and processed using the Mega Win software that accompanies the Finnish ME6000, with a sampling frequency of 1,000 Hz. The raw electromyography (EMG) was intercepted, selected for root mean square data conversion, smoothed, etc., and the root mean square amplitude (RMS) was selected as the EMG index. Amplitude (RMS) was selected as the maximum value. Between-group comparisons were made using a processing analysis with large individual differences in EMG indicators, and the rate of change of RMS was calculated = (post-measurement value - pre-measurement value)/pre-measurement value × 100% for between-group comparisons.

### 2.6 Blood markers

Blood samples were collected early in the morning on the first day of training and the day after the experiment. Subjects were fasted for a minimum of 12 h before blood collection. Five ml of venous blood was collected on an empty stomach to determine fasting blood glucose (FBG), fasting insulin (FINS), glycated hemoglobin (HbA1C) and total cholesterol (TC), triglycerides (TG), high-density lipoprotein cholesterol (HDL-C) and low-density lipoprotein cholesterol (LDL-C). The professional medical staff at Dalian Fifth People’s Hospital took all the blood.

### 2.7 Blood pressure and heart rate variability (HRV) tests

A professional blood pressure measuring instrument (HEM-7211 Omron electronic blood pressure monitor) was used to measure the systolic blood pressure (SBP) and diastolic blood pressure (DBP) of the subjects in the morning under fasting conditions. During the test, the right arm was naturally relaxed and straightened. The subject’s arm, blood pressure monitor, and heart position were kept at the same height. Three consecutive measurements were taken (the middle value was taken), and the test results were expressed in millimeters of mercury (mmHg).

For the HRV test, the subjects wore a Polar Team2 Team Heart Rate Telemetry manufactured in Finland and were staying in quiet condition for 5 min. Real-time monitoring of the subjects’ quiet heart rate (RHR) and whole heart rate was performed, and the heart rate was measured from 8:30 a.m. to 9:20 a.m. and collected for 10 min. Time domain indicators of HRV were calculated using the Kubios HRV software: standard deviation of R-R intervals (SDNN) for global HRV changes, and the root mean square of the difference between the adjacent R-R intervals (RMSSD) for the parasympathetic drive. Frequency-domain indicators were high-frequency power (HF) for parasympathetic modulation, low-frequency power (LF) for predominantly sympathetic activity, and the ratio of high-frequency power to low-frequency power (LF/HF) for both sympathetic and parasympathetic homeostasis.

### 2.8 Mathematical and statistical method

Test metrics were collected and organized using Excel statistical software and SPSS 22.0 statistical software. All data were expressed as mean ± standard deviation (M ± SD). The Kolmogorov-Smirnov test was used to assess normally distributed variables. The one-way variance was used for analysis between groups; paired samples *t*-test was used for analysis within groups. *p <* 0.05 indicates statistical significance.

## 3 Results

### 3.1 Body composition index

Pre and post-group comparisons revealed that body fat percentage, fat content, and waist-to-hip ratio were highly significantly lower (*p <* 0.01), and both lean mass and muscle mass were highly significantly greater (*p <* 0.01) in BFRT group. A between-group comparison revealed that waist-to-hip ratio was significantly lower (*p <* 0.01), lean body and muscle mass were significantly greater (*p <* 0.05), and percent body fat was significantly less (*p <* 0.05) in BFRT group compared to the CON group. Compared to RT group, the subjects in BFRT group showed a significant decrease in waist-to hip-ratio (*p <* 0.01), and significant up-regulations in lean mass and muscle mass (*p <* 0.05) (Table 3).

**TABLE 3:** Changes in body composition indicators of the subjects before and after the experiment (M ± SD).

Target	CON group		RT group		BFRT group	
	Baseline	12 weeks post	Baseline	12 weeks post	Baseline	12 weeks post
Weight(kg)	76.12 ± 6.78	76.73 ± 7.15	74.98 ± 5.49	74.74 ± 5.71	78.28 ± 6.97	77.72 ± 6.87
Lean Mass (kg)	57.76 ± 3.81	56.84 ± 4.02	57.10 ± 3.36	56.92 ± 3.47	58.94 ± 4.38	60.84 ± 3.98**^ab^
BF (%)	23.38 ± 2.91	24.79 ± 2.80	23.81 ± 2.79	23.57 ± 2.52	24.73 ± 2.89	21.53 ± 2.36**^a^
Body Fat Content (kg)	17.81 ± 3.65	19.36 ± 3.71	17.93 ± 3.25	17.73 ± 3.01	19.50 ± 3.47	16.88 ± 3.29**
WHR	0.87 ± 0.02	0.87 ± 0.02	0.89 ± 0.03	0.87 ± 0.01	0.88 ± 0.02	0.84 ± 0.02**^aabb^
Muscle Mass(kg)	53.40 ± 3.46	52.31 ± 3.79	52.79 ± 3.08	52.56 ± 3.20	54.42 ± 4.02	56.23 ± 3.41**^ab^

BF (%), body fat percentage; WHR, waist-to-hip ratio; *, denotes the comparison between the pre and post-experimental groups themselves, *p < 0.05, **p < 0.01.

^a^represents the between-group comparison between the post-experiment group and the CON, group. ^a^ *p <* 0.05, ^aa^ *p <* 0.01.

^b^depicts the post-experiment between-group comparison and RT. ^b^ *p <* 0.05, ^bb^ *p* < 0.01.

### 3.2 Neuromuscular activation

When comparing the neuromuscular activation indexes within groups after the 12-week experiment, the changes in the lateral femoral muscle RMS of the knee, medial femoral muscle RMS of the knee and biceps RMS in the CON and RT groups compared to the pre-experimental period were not significant (*p >* 0.05); The medial femoral muscle RMS of the knee and the biceps RMS were significantly improved (*p <* 0.01) and the lateral femoral muscle RMS of the knee was significantly improved (*p <* 0.05) after the experiment in BFRT group compared to the pre-experimental period (Table 4).

**TABLE 4:** Changes in RMS maxima within the group before and after the experiment for obese college students (M ± SD).

Target	CON group		RT group		BFRT group	
	Baseline	Post 12 weeks	Baseline	Post 12 weeks	Baseline	Post 12 weeks
Lateral Femoral Muscle RMS (uv)	380.63 ± 211.64	294.13 ± 123.12	398.11 ± 162.44	450.67 ± 191.35	286.44 ± 95.94	468.00 ± 227.26*
Medial Femoral Muscle RMS (uv)	434.63 ± 154.73	365.38 ± 100.85	382.22 ± 134.95	401.89 ± 123.15	419.56 ± 156.21	586.44 ± 219.08**
Biceps RMS (uv)	1,025.63 ± 601.51	833.1 ± 315.38	556.55 ± 298.06	642.56 ± 270.03	908.22 ± 417.54	1,567.33 ± 786.93**

*denotes the comparison between the pre and post-experimental groups themselves, *p < *0.05*, **p < 0.01.

When comparing the RMS change rates between groups after 12 weeks of the experiment, the rates of change of the lateral femoral muscle RMS of the knee, medial femoral muscle RMS of the knee, and biceps RMS in RT group and CON group were not significant (*p >* 0.05); the rates of change of the lateral femoral muscle RMS of the knee, medial femoral muscle RMS of the knee, and biceps RMS in BFRT group compared with the CON group were significantly higher (*p <* 0.01); the rates of change of the lateral femoral muscle RMS of the knee, medial femoral muscle RMS of the knee, and biceps RMS were significantly higher in BFRT group compared with the RT group (*p <* 0.05) (Table 5).

**TABLE 5:** Comparison of the rate of change of RMS between groups after the experiment for obese college students (M ± SD).

Target (%)	CON	RT	BFRT
Lateral Femoral Muscle RMS	−8.62 ± 43.70	18.56 ± 36.93	77.78 ± 48.28^aab^
Medial Femoral Muscle RMS	−7.50 ± 36.23	9.11 ± 22.03	40.44 ± 18.07^aab^
Biceps RMS	−4.00 ± 43.47	31.10 ± 37.87	80.22 ± 38.58^aab^

RMS, rate of change = (post-test value - pre-test value)/pre-test value × 100%.

^a^represents the between-group comparison between the post-experiment group and the CON, group. ^a^ *p < 0.05*, ^aa^ *p* < *0.01*.

^b^depicts the post-experiment between-group comparison and RT. ^b^ *p < 0.05*, ^bb^ *p* < *0.01*.

### 3.3 Muscle fitness

When compared within groups after 12 weeks of the experiment, there was no significant change in all indexes in CON group (*p >* 0.05); the knee 180° extensor endurance ratio and elbow 180° flexor endurance ratio in RT group had significant improvements (*p <* 0.01); the knee extensor isometric PT, elbow flexor isometric PT, knee 180° extensor PP, and elbow 180° flexor PP in BFRT group had significant improvements after the experiment (*p <* 0.01). The knee 180° extensor endurance ratio and elbow 180° flexor endurance ratio were all significantly improved in BFRT group (*p < 0.01*). The knee 180° extensor endurance ratio and elbow 180° flexor endurance ratio were significantly higher in RT group compared to CON group (*p <* 0.01). The knee extensor isometric PT, elbow flexor isometric PT, knee 180° extensor PP, elbow 180° flexor PP, knee 180° extensor endurance ratio, and elbow 180° flexor endurance ratio were all significantly higher in BFRT group compared to CON group (*p <* 0.01). The knee extensor isometric PT, elbow flexor isometric PT, knee 180° extensor PP, and elbow 180° flexor PP were all significantly higher in BFRT group compared to RT group (*p < 0.01*), and the knee 180° extensor endurance ratio was higher compared to the RT group (*p <* 0.05) (Table 6).

**TABLE 6:** Changes in muscle fitness of obese college students before and after the experiment.

Target	CON group		RT group		BFRT group	
	Baseline	Post 12 weeks	Baseline	Post 12 weeks	Baseline	Post 12 weeks
Knee extensor isometric PT(Nm)	171.00 ± 45.11	161.38 ± 34.47	174.08 ± 46.58	174.22 ± 33.95	172.03 ± 34.91	217.96 ± 24.52**^aabb^
Elbow flexor isometric PT(Nm)	40.83 ± 11.23	37.65 ± 7.72	42.28 ± 13.25	47.01 ± 8.58	49.02 ± 8.78	65.57 ± 15.28**^aabb^
Knee 180°extensor PP(W)	279.40 ± 77.00	248.74 ± 59.19	292.06 ± 98.51	286.51 ± 84.69	349.36 ± 57.22	449.91 ± 89.03**^aabb^
Elbow 180°flexor PP(W)	53.30 ± 19.46	52.44 ± 22.60	56.63 ± 17.67	56.40 ± 15.31	65.61 ± 25.76	88.87 ± 31.64**^aabb^
Knee 180°extensor endurance ratio (ER) (%)	73.50 ± 6.87	71.00 ± 6.70	76.33 ± 5.61	84.11 ± 3.62**^dd^	75.67 ± 4.98	90.44 ± 5.55**^aab^
Elbow 180°flexor endurance ratio (ER) (%)	68.13 ± 9.88	66.63 ± 6.84	69.89 ± 6.85	77.56 ± 6.39**^dd^	72.33 ± 7.94	82.00 ± 9.17**^aa^

*denotes the comparison between the pre and post-experimental groups themselves, *p < *0.05*, **p < *0.01*.

^a^represents the between-group comparison between the post-experiment group and the CON, group. ^a^ *p < 0.05*, ^aa^ *p* < *0.01*.

^b^depicts the post-experiment between-group comparison and RT. ^b^ *p < 0.05*, ^bb^ *p* < *0.01*.

### 3.4 Glucose metabolism index

FINS was significantly lower in RT group before and after the within-group comparison (*p < 0.05*); There were significant down-regulations in FBG, FINS, HbA1C, and HOMA-IR in BFRT group (*p < 0.01*). A between-group comparison revealed that the FBG and HOMA-IR were significantly lower in BFRT group compared to the RT group (*p < 0.01*). Compared to the CON group, the HbA1C and HOMA-IR values were significantly lower in BFRT group (*p < 0.05*) and the FBG values were significantly lower (*p < 0.01*) (Table 7).

**TABLE 7:** Changes of glucose metabolism indexes in subjects before and after the experiment.

Target	CON group		RT group		BFRT group	
	Baseline	Post 12 weeks	Baseline	Post 12 weeks	Baseline	Post 12 weeks
FBG(mmol/L)	4.85 ± 0.43	4.92 ± 0.50	4.97 ± 0.42	4.95 ± 0.39	5.03 ± 0.60	4.31 ± 0.17**^aabb^
FINS(uU/mL)	9.04 ± 0.49	9.07 ± 0.41	9.16 ± 0.40	9.12 ± 0.41*	9.04 ± 0.65	8.83 ± 0.64**
HbA1C(%)	5.30 ± 0.58	5.31 ± 0.52	4.94 ± 0.67	4.92 ± 0.68	5.10 ± 0.54	4.68 ± 0.40**^a^
HOMA-IR	1.98 ± 0.26	1.99 ± 0.28	2.03 ± 0.26	2.01 ± 0.24	2.03 ± 0.35	1.70 ± 0.18**^abb^

HOMA-IR: the insulin resistance index, calculated from the fasting blood glucose level and insulin concentration, the formula is as follows: HOMA-IR = Insulin [μU/mL] × Glucose [mmol/L]/22.5; * denotes the comparison between the pre and post-experimental groups themselves, *p < 0.05, **p < 0.01.

^a^represents the between-group comparison between the post-experiment group and the CON group, ^a^ *p < 0.05*, ^aa^ *p < 0.01*.

^b^depicts the post-experiment between-group comparison and RT, ^b^ *p<0.05*, ^bb^ *p < 0.01*.

### 3.5 Lipid metabolism index

In the pre and post-group comparisons, subjects in RT group showed significant changes in HDL-C and LDL-C (*p <* 0.05), and subjects in BFRT group had a significant decrease in TC (*p <* 0.05), a significant increase in HDL-C (*p <* 0.01) and a significant decrease in LDL-C (*p <* 0.01). A between-group comparison revealed that there were a significant increase in HDL-C (*p <* 0.05) in RT group compared to the CON group, and a significant increase in HDL-C (*p <* 0.05) in BFRT group compared to RT group, and a significant decrease in LDL-C (*p <* 0.05) as well as a highly significant increase in HDL-C (*p <* 0.01) in BFRT group compared to CON group (Table 8).

**TABLE 8:** Changes of lipid metabolism indexes in subjects before and after the experiment (M ± SD).

Target	CON group		RT group		BFRT group	
	Baseline	Post 12 weeks	Baseline	Post 12 weeks	Baseline	Post 12 weeks
TG (mmol/L)	0.91 ± 0.24	0.94 ± 0.26	1.04 ± 0.40	1.07 ± 0.35	1.00 ± 0.38	1.00 ± 0.38
TC (mmol/L)	4.13 ± 0.34	4.17 ± 0.31	3.98 ± 0.60	3.96 ± 0.59	4.25 ± 0.44	4.22 ± 0.44*
HDL- C(mmol/L)	1.14 ± 0.08	1.13 ± 0.73	1.23 ± 0.15	1.28 ± 0.13*^a^	1.28 ± 0.19	1.41 ± 0.13**^aab^
LDL- C(mmol/L)	2.63 ± 0.50	2.64 ± 0.50	2.44 ± 0.58	2.40 ± 0.55*	2.28 ± 0.70	2.03 ± 0.61**^a^

*denotes the comparison between the pre and post-experimental groups themselves, *p < *0.05*, **p < *0.01*.

^a^represents the between-group comparison between the post-experiment group and the CON, group. ^a^ *p < 0.05*, ^aa^ *p* < *0.01*.

^b^depicts the post-experiment between-group comparison and RT. ^b^ *p < 0.05*, ^bb^ *p* < *0.01*.

### 3.6 HRV and blood pressure

In the pre and post-group comparisons, SBP was significantly lower (*p < 0.05*), RMSSD and HF were significantly higher (*p < 0.05*), and SDNN and LF were significantly higher (*p < 0.01*) in BFRT group. Comparison between groups revealed that the BFRT group had significantly lower SBP (*p < 0.05*), significantly higher RMSSD and HF (*p < 0.05*), and significantly higher SDNN and LF (*p < 0.01*) compared with the CON group; the BFRT group had significantly lower SBP (*p < 0.05*) and significantly higher SDNN and RMSSD (*p < 0.05*) compared with the RT group (*p < 0.05*), and LF had a highly significant increase (*p < 0.01*) (Table 9).

**TABLE 9:** Changes in physical function indexes of the subjects before and after the experiment (M ± SD).

Target	CON group		RT group		BFRT group	
	Baseline	Post 12 weeks	Baseline	Post 12 weeks	Baseline	Post 12 weeks
RHR(/min)	72.35 ± 12.78	71.58 ± 13.67	67.92 ± 12.54	68.57 ± 11.03	69.15 ± 11.35	70.56 ± 10.41
SBP (mm/Hg)	117.56 ± 4.85	118.16 ± 5.27	119.54 ± 4.79	118.69 ± 5.13	119.23 ± 4.82	115.85 ± 5.60^*ab^
DBP (mm/Hg)	67.58 ± 7.56	68.21 ± 6.89	69.82 ± 5.87	69.51 ± 6.21	68.53 ± 6.57	68.14 ± 5.68
SDNN (ms)	14.92 ± 2.95	15.38 ± 3.27	16.25 ± 3.47	17.02 ± 2.81	15.29 ± 2.84	21.38 ± 4.67**^aab^
RMSSD (ms)	9.75 ± 1.84	9.26 ± 1.96	8.82 ± 1.86	9.31 ± 1.42	9.57 ± 1.25	14.28 ± 4.16*^ab^
LF (ms^2^)	128.67 ± 54.82	129.54 ± 68.45	131.54 ± 56.42	143.69 ± 48.75	122.86 ± 38.61	295.75 ± 61.76**^aabb^
HF (ms^2^)	18.64 ± 7.24	17.89 ± 5.94	17.68 ± 6.78	21.53 ± 9.24	20.75 ± 8.73	49.78 ± 19.82*^a^
LF/HF	7.34 ± 3.65	7.59 ± 3.21	8.56 ± 3.35	7.54 ± 3.94	6.84 ± 3.72	8.35 ± 6.28

RHR, quiet heart rate; SBP, systolic blood pressure; DBP, diastolic blood pressure; * denotes the comparison between the pre and post-experimental groups themselves, *p < *0.05*, **p < *0.01*.

^a^represents the between-group comparison between the post-experiment group and the CON, group. ^a^ *p < 0.05*, ^aa^ *p* < *0.01*.

^b^depicts the post-experiment between-group comparison and RT. ^b^ *p < 0.05*, ^bb^ *p* < *0.01*.

## 4 Discussion

The aim of this study was to investigate the effects of BFR combined with low-intensity RT training on cardiovascular risk factors in obese people. The main results showed that BFR combined with low-intensity RT training resulted in a significant decrease in body fat percentage and waist-to-hip ratio, a significant increase in lean mass and muscle mass, and a muscle-building and fat-loss effect in obese subjects, as well as an enhancement of peak torque, peak power, and endurance ratio of muscle-fit knee extensors and flexors; and a significant increase in RMS of medial femur, lateral femur, and biceps brachii muscles. In addition, subjects’ DBP was significantly reduced, and heart rate variability indexes RMSSD, HF, SDNN, and LF were significantly upregulated. While the glycolipid metabolism changes HbA1C, HOMA-IR and FBG were significantly downregulated. Meanwhile, LDL-C was significantly decreased and HDL-C was significantly increased. In conclusion, BFR combined with low-intensity RT training can effectively reduce body fat, increase muscle mass, improve neuromuscular activation, enhance muscle strength and endurance, and thus improve abnormal glucose-lipid metabolism and enhance cardiac autoregulation.

Obesity is associated with a high risk of cardiovascular disease. It has been suggested that multiple neurohumoral, metabolic, and hemodynamic components are potential impact factors between obesity and CVD, but the exact mechanisms are still not completely understood (Wormser et al., 2011; Yusuf and Anand, 2011; Khan et al., 2018). Body fat percentage and waist-to-hip ratio are accurate indicators for assessing the impact of obesity on CVD (Myint et al., 2014). Epidemiological and clinical studies over the past 30 years have demonstrated a strong association between visceral fat ectopic deposition and the development of clinical syndromes characterized by atherogenic dyslipidemia, dyslipidemia (hypertriglyceridemia and reduced HDL), hyperinsulinemia/glucose intolerance, hypertension, reduction in heart rate variability, atherosclerosis, and adverse cardiac remodeling/heart failure (Neeland et al., 2018). In the present study, it has been shown that these components related to metabolism are independent risk factors for CVD (Bozkurt et al., 2016; ESC Committee for Practice Guidelines and ESC National Cardiac Societies, 2019; Katta et al., 2021).

However, the effect of decreasing body mass index (BMI) or weight loss on the risk of CVD remains uncertain. Several studies have reported that lower BMI is associated with a reduced risk of CVD in younger adults (Choi et al., 2018), but others in older adults and patients with chronic disease have found a non-significant association between weight loss and the progression and mortality of CVD (Zheng et al., 2017; Kjøllesdal et al., 2020), and some even demonstrated that weight loss is associated with an increased risk of CVD and/or its mortality (Strand et al., 2017; Doehner et al., 2020). One possible reason for the uncertain association between weight loss and a protective effect on the primary prevention of CVD may be that reductions in fat mass and reductions in muscle mass have differential effects on the development of CVD. In other words, a reduction in adipose tissue mass is positively associated with the risk of developing CVD, while a reduction in muscle mass is negatively associated with the risk of developing CVD (Kim et al., 2022), implying that one of the major reasons for the unexpected negative effects of weight loss on cardiovascular disease could be due to the loss of more muscle mass than adipose tissue mass, which suggests that weight loss alone may not be necessarily beneficial in the prevention of cardiovascular disease (Kim et al., 2022). Additionally, recent studies have shown that weight loss induced by caloric restriction can exacerbate sarcopenia by reducing both lean body mass and muscle strength. In contrast, increasing moderate-intensity exercise maintains lean body mass and preserves muscle strength in the face of weight loss (Brennan et al., 2022). In general, aerobic exercise greatly improves cardiorespiratory fitness and cardiometabolic variables, whereas resistance exercise primarily affects muscle strength and has a positive impact on body composition (e.g., muscle mass) (Schroeder et al., 2019). Moreover, RT may be associated with adverse cardiovascular responses (e.g., altered hemodynamics, and ventricular arrhythmias) (Kambič et al., 2019).

A training load of >75% 1-RM is required in healthy adults in order to achieve optimal improvements in muscle hypertrophy and muscle strength. However, the recommended load for patients with coronary artery disease is to be <30% 1-RM, which is insufficient to induce gains in isometric strength and hypertrophy (Bjarnason-Wehrens et al., 2004; American College of Sports Medicine position stand, 2009; Piepoli et al., 2011). Thus, BFR as a novel method for promoting muscle growth and improving muscle function by inducing muscle hypertrophy and strength at low-to-moderate training intensities, simulated moderate-to-high-load intensity training, is relatively safe and has been applied in the training of patients with cardiovascular disease. BFR in healthy adults combined with RT may also improve muscle strength and hypertrophy at lower loads while minimizing adverse systemic cardiovascular outcomes (Kambič et al., 2019). Thus, in the present study, BFR in combination with low-intensity RT was used to not only reduce fat mass, body fat percentage and waist-to-hip ratio in obese individuals but also increase lean mass and muscle mass for fat loss and muscle gain.

Increased muscle mass is associated with a reduced risk of developing CVD. Increasing muscle mass through regular exercise and intentional weight loss may help to reduce total blood volume, volume per beat, preload, and left ventricular filling pressures, with beneficial effects on cardiovascular health (Elagizi et al., 2018). Studies have shown that the benefits of increased muscle mass on cardiovascular health are maintained even in individuals with an increase in body mass index or weight gain (Li et al., 2022), suggesting the importance of muscle mass, as opposed to simply lowering BMI, in preventing the onset of cardiovascular disease. Modulation of neural factors associated with muscle strength is the primary mechanism for strengthening muscle strength. Electromyography can reflect the effects of neural entrainment and other factors during muscle contraction. Increases in muscle force are related to the excitability of motor units as well as the frequency of nerve recruitment of muscle signals. Root mean square (RMS) amplitude refers to the effective value of the neural discharge, which is linked to motor unit recruitment and synchrony of excitatory rhythms and may be a good response depending on the degree of neuromuscular activation (Coratella et al., 2021). Some studies have found that all neuromuscular activation is significantly increased following RT (Sousa et al., 2017). In addition, the present study demonstrated that the rate of change in RMS in muscle tested in the BFRT group was positive, and the RMS increase in BFRT group was significantly greater than that in CON group and RT group, suggesting that the 12-week BFR training with low-intensity RT triggers an increase in motor neuron control of fast muscle fibers, an increase in neural discharge and a significant increase in neuromuscular activation.

Maximal strength, rapid strength and muscle endurance are the common indicators of muscle strength response. Peak torque (PT), peak power (PP) and endurance ratio are well-acknowledged indicators for evaluating maximum muscle strength, the output of maximum muscle fiber force during exercise, and muscle endurance, respectively (Knapik et al., 1996; Yáñez-Silva et al., 2017; Osawa et al., 2018; Akınoğlu and Kocahan, 2020). Weightlifters showed significant improvements in lower limb PT levels and muscle circumference after 6 weeks of 30% 1RM BFR training with low-intensity RT (Luebbers et al., 2014). In healthy residents, the maximal strength of the lower extremity extensor increased significantly after 12-week 20% 1RM BFR training with low-intensity RT (Luebbers et al., 2014; Lixandrão et al., 2015). Our current research is consistent with most of the previous results. Obese subjects in BFRT group showed significant improvement in knee and elbow PT and increased maximal muscle strength. The elongation of the knee joint and elbow flexor maximal strength was improved, which provided the basis for the rapid strength improvement. Yasuda et al. (2005) confirmed that BFR training significantly increases the cross-section volume of type II fibers by 27.6%, significantly higher than the cross-section volume of type I fibers, which may increase the rapid strength of muscle fiber. In our study, knee extensor PP and elbow flexor PP were significantly elevated in BFRT group, while motor unit recruitment increased, and muscle strength enhanced rapidly at higher thresholds in BFRT group. This may be due to reduced oxygen supply to muscle fibers at the junction as a result of BFR training, leading to a significant accumulation of metabolites, which in turn induce muscle fatigue through cross-bridge cyclic inhibition, resulting in the recruitment of high-threshold exercise units and triggering the activation and enhancement of fast muscle fibers.

The endurance ratio is an index of muscle endurance response and fatigue resistance. Previous studies have demonstrated that BFR training combined with low-intensity RT can improve muscular endurance in subjects (Henrique et al., 2019; Perera et al., 2022). The results of the current study showed that the knee extensor, and elbow flexor endurance ratios in the obese group significantly increased after the experiment in both RT and BFRT groups compared with the pre-experimental group, with the ratio of knee extensor endurance in BFRT group being greater than the ratio in RT group, and the effect of BFR training combined with low-intensity RT in improving muscular endurance in the obese group was superior. This may be result of upregulated metabolites induced by BFR training and stimulated nervous system caused by the mobilization of fast muscle fibers via neurofeedback pathways to improve coordinated regulation, which diffuses central nervous excitation and alter motor neuron recruitment to make recruitment more efficient, thereby increasing cortico-spinal excitability and increasing its ability to adapt to the intensity of exercise during training (Næss-Schmidt et al., 2017).

Muscle strength (MusS) has been shown to have independent and joint relationships with markers of obesity and blood pressure and atherosclerosis. A cross-sectional study found that higher levels of MusS in males were associated with lower triglycerides (up to 0.33-fold lower) and HOMA-IR (up to 0.35-fold lower), and higher HDL cholesterol (up to 5.2 mg/dL). Higher levels of MusS were associated with lower SBP and DBP (up to −10.2 mmHg), as well as HOMA-IR (up to 0.27-fold) in women. Furthermore, higher levels of MusS were inversely associated with triglycerides and HOMA-IR in obese males and DBP only in obese females. These population-based studies confirm the findings of clinical studies and suggest that in obese individuals, higher levels of MusS may contribute to reduced cardiometabolic risk in adults (de Lima et al., 2022).

Consistence with previous studies (Lira et al., 2010; Saatmann et al., 2021; Alizadeh Pahlavani, 2022), the current study showed significantly reduced TC and LDL-C levels as well as elevated HDL-C levels in BFRT group while improving muscle strength following BFR training. BFR is considered with the effect of accelerating lipolysis rate and ameliorating abnormal lipid metabolism by promoting hormone secretion. While RT remains controversial in reports of insulin sensitivity, values for the insulin resistance index (HOMA-IR) are primarily related to FBG and FINS concentrations in the body. BFR in combination with RT has been shown to improve insulin sensitivity (Kambič et al., 2019). These findings agree with our findings that the BFRT group significantly reduced insulin, HbA1C, FBG, and FINS, as well as HOMA-IR levels in obese individuals, suggesting that BFR training can effectively improve the level of glucose metabolism in subjects, by improving insulin resistance and thereby reducing the occurrence of CVD events. A similar beneficial effect of resistance exercise with higher loads has also been reported in type 2 diabetes (Jorge et al., 2011; Kadoglou et al., 2013). Patients with type 2 diabetes have shown a significant decrease in HOMA-IR following 6 months of RT at an exercise intensity of 60%–80% 1-RM (Kadoglou et al., 2013). In contrast, in patients with type 2 diabetes of a similar age, HOMA-IR showed no significant change after 12-week RT (Jorge et al., 2011), indicating that the reduction in HOMA-IR may be intensity-and time-dependent.

Autonomic nervous system (ANS) dysfunction in people with obesity is strongly associated with the onset of cardiovascular risk (Russo et al., 2021). Heart rate variability (HRV) is a valid indicator for the assessment of ANS, and a reduction in HRV significantly increases cardiovascular mortality (Barthelemy et al., 2022). While most studies of BFR have focused on muscle effects, it is important to note that resistance exercise and aerobic exercise in BFR can lead to elevated heart rate and blood pressure with a greater increase than that seen with exercise performed at similar intensities in the absence of BFR (Hackney et al., 2012). RMSSD is an index for assessing parasympathetic activity. In a study enrolling 17 healthy older adults who performed high-intensity resistance exercise for 12 weeks, RMSSD showed no significant change in the resistance exercise group (Millar et al., 2012). While, by the findings of Junior et al. (2019), our results showed that the time domain indicators of HRV, SDNN and RMSSD, were significantly elevated in BFRT group, and the reason for this discrepancy could be attributed to the different forms and amount of exercise, and the subject variance. LF closely represents sympathetic regulation, and HF is an indicator of vagal regulation (Zhao et al., 2022). In the present study, HF and LF were also found to be significantly elevated in BFRT group, with enhanced sympathetic and parasympathetic excitability, possessing improved regulation relative to resistance training alone. High SBP is a leading cause of death and disability worldwide (Naci et al., 2019), and moderate to high-intensity resistance exercise can effectively lower blood pressure. One possible explanation for the significant reduction in SBP in BFRT group in the present study is that BFR training results in the production of large amounts of vascular endothelial growth factor (VEGF) and endothelial nitric oxide synthase (eNOS), which in turn promotes vascular nitric oxide (NO) production and has a vasodilatory effect in the prevention and treatment of hypertension, which may reduce the incidence of CVD (Zhang et al., 2022).

## 5 Conclusion

In conclusion, our results suggest that BFR training with low-intensity RT is effective not only in improving muscle strength and body fat composition for muscle gain and fat loss, but the training effect is similar to that of a training load of >75% 1-RM. It also improves the abnormalities of glucolipid metabolism in the obese college student body. In addition, BFR training with low-intensity RT is relatively safe with a beneficial effect on the hemodynamic response and enhances cardiac autonomic regulation. Thus, it may serve as an additional exercise option to safely and effectively improve metabolic dysfunction and overall health caused by high adiposity and low muscle mass. However, the current study suggests that future studies are still required to assess organismal metabolic function on larger samples and longer training durations. Clarify the effects of BFR training on different health parameters may reduce the aggregation of cardiovascular risk factors, and thus reduce the incidence of cardiovascular disease.

## 6 Limitations and strengths

Obesity is gradually rejuvenating and has become one of the urgent problems in the society. BFR training, as one of the training methods for muscle building and fat loss, has been less studied in the obese population. Therefore, this study comprehensively explored the effect of BFR training combined with low-intensity resistance training to enhance muscle building and fat loss in college obese population through the measurement of obesity, muscle fitness and cardiovascular risk factors, and then improved cardiovascular risk factors, which provided theoretical and practical guidance for the improvement of the health status of young obese population. However, due to the diversity of the population to which BFR is applied, there are limitations in this study in choosing only young obese people, and it is possible to apply BFR training to a wider range of people in the future, so as to contribute to the fitness of the general public.

## Data Availability

The raw data supporting the conclusion of this article will be made available by the authors, without undue reservation.
